# Understanding Hydration Transitions of CaBr_2_

**DOI:** 10.1021/acs.cgd.4c01522

**Published:** 2025-03-27

**Authors:** Michaela C. Eberbach, Aleksandr I. Shkatulov, Paul Tinnemans, Hendrik P. Huinink, Hartmut R. Fischer, Olaf C. G. Adan

**Affiliations:** † Eindhoven University of Technology, Den Dolech 2, 5600 MB Eindhoven, The Netherlands; ‡ EIRES, Horsten 1, 5612 AX Eindhoven, The Netherlands; § Iberian Center for Research in Energy Storage, CIIAE, Polígono 13, Parcela 31, “El Cuartillo”, 10004 Cáceres, Spain; ∥ Radboud University, Houtlaan 4, 6525 XZ Nijmegen, The Netherlands; ⊥ TNO Materials Solutions, High Tech Campus 25, 5656 AE Eindhoven, The Netherlands

## Abstract

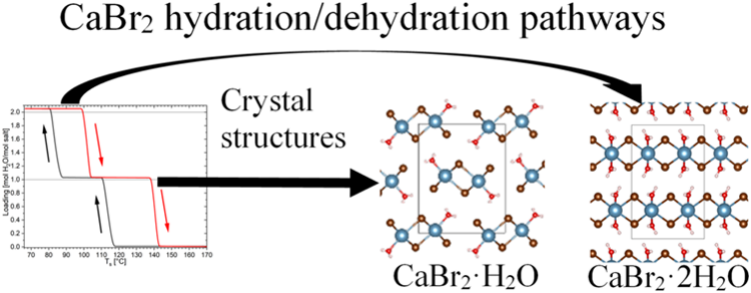

Due to climate change and the energy transition, energy storage applications are being studied and developed. One energy storage application is a heat storage battery, which needs materials that can store and release heat with high energy storage capacity. One such material is a salt hydrate. The hydration pathways of salt hydrates can have different numbers of steps. There are salts with single-hydrate steps like for CuCl_2_ (0–2) and LiBr (0–1) and multihydrate steps like for MgCl_2_ (0–2–4–6) and SrCl_2_ (0–1–2–6). Additionally, there are also salts with complex hydration–dehydration pathways like for CaCl_2_ (0–1/3–2–1–0). Little is known about the hydrate steps of CaBr_2_. The crystal structures of the CaBr_2_ nona-, hexa-, and anhydrate are known, but there are no intermediate steps and conditions for these transitions. The hexahydrate and anhydrate have the same structure as CaCl_2_ except for the unit cell size due to the different anions. Additionally, the equilibria were determined for the hexa-, tetra-, and dihydrate transitions. However, the intermediate steps are debated. The hydrates 3, 1.5, 1, and 0.5 were all proposed but are disputed and not verified. Therefore, the hydration and dehydration pathways of CaBr_2_ from the anhydrate to the dihydrate and back were examined in this study for both the bulk salt and the confinement of mesoporous silica gels. The kinetic phase transition onsets and equilibrium lines were measured for the bulk salt. Powder X-ray diffractograms were used to ensure that the same structures were formed every time during hydration and dehydration. Single-crystal analysis was used to determine the crystal structures of the hydrates. These experiments showed only a stable monohydrate phase between the anhydrate and dihydrate during hydration and dehydration. Furthermore, the dihydrate has the same crystal structure as the dihydrate of CaCl_2_ except for the size, while the monohydrate differs from the CaCl_2_ monohydrate. Additionally, the composites’ kinetic onsets and powder diffractograms were measured, which showed that CaBr_2_ performs the same hydrate steps in confinement as in bulk form.

## Abstract 2

The hydration and dehydration steps of the salt hydrate CaBr_2_ were determined with their formation and stable conditions together with the crystal structures of the mono- and dihydrate.

## Introduction

Salt hydrates are promising materials for thermal energy storage (TES)^1−5^ due to their high energy density and the ability to store energy without loss during hydration transitions.^1,6−8^ For thermochemical energy storage (TCES), the salt hydrate has to undergo hydration reactions with water vapor to perform a solid–solid phase transition from one hydrate to another.^9^ Salt hydrates can have a single hydration step like LiCl,^9^ LiBr,^10^ and CuCl_2_^9^ or it can have multiple hydration steps like MgCl_2_,^9,11^ SrCl_2_,^12^ and SrBr_2_.^13,14^ Usually, these multihydrate steps are performed one after the other and the same way in reverse during dehydration. There are exceptions like CaCl_2_,^15,16^ which have path-dependent hydration–dehydration steps. In the case of CaCl_2_, the hydration path goes from the anhydrate via the tritohydrate to the dihydrate, while the dehydration steps go from the dihydrate via the monohydrate to the anhydrate. So, during hydration, only the tritohydrate is observed, while the monohydrate is only detected during dehydration at lower water vapor pressures.^15,16^ This path dependency could be linked to kinetic hindrances between the hydrate steps due to their crystal structures.^16^

For CaBr_2_, many different hydrates are reported. In the Gmelin handbook, the hydrates 6, 4, 3, 2, 1.5, 1, and 0.5 are mentioned.^17^ However, the existence of the 0.5-hydrate is disputed as the lowest hydrate during dehydrations.^18^ Furthermore, in dehydration experiments by Paulik et al. in 1979, the water content correlating with the trihydrate could also be the result of solid phases in contact with a saturated solution of CaBr_2_ instead of the pure solid hydrate.^19^ Of these different reported hydrates, only four crystal structures are found in crystal databases: two anhydrate polymorphs, the hexahydrate, and the nonahydrate, as indicated in the right columns of Table 1.

**Table 1:** Known Crystal Structures of the CaCl_2_ and CaBr_2_ Hydrates Taken from refs (16,30−31,32,33,34,40)

	CaCl_2_			CaBr_2_		
hydrate	space group	symmetry	*V* [nm^3^]	space group	symmetry	*V* [nm^3^]
anhydrate	*Pnnm*	orthorhombic	0.169	*Pnnm*	orthorhombic	0.196–0.202
tritohydrate	*Pnma*	orthorhombic	0.943			
monohydrate	*Pmmn*	orthorhombic	0.185			
dihydrate	*Pbcn*	orthorhombic	0.531			
α-tetrahydrate	*P*-1	triclinic	0.331			
β-tetrahydrate	*P*2	monoclinic	1.060			
γ-tetrahydrate	*P*2	monoclinic	0.391			
hexahydrate	*P*321	trigonal	0.212	*P*321	trigonal	0.232
nonahydrate				*P*2_1_	monoclinic	1.229

Regarding the hydration and dehydration steps determined for CaBr_2_, there are conflicting sources in the literature. An examination of phase transition equilibria by Bassett et al. showed the transitions between the hydrates 6, 4, and 2.^20^ The newer articles suggest the hydration steps 0–6^2,13,21,22^ and 6–9,^22^ but give no indication of intermediate steps. Furthermore, no transitions to or from the nonahydrate could be discovered. Similarly, no reported pathways between anhydrate and dihydrate could be found.

While many articles with the CaBr_2_ being used in combination with other salts^23−29^ were identified, no literature was found on the hydration–dehydration steps of this salt in confinement.

By comparing the structures of the known CaBr_2_ hydrates^30−33^ with the corresponding hydrates of CaCl_2_ hydrates^16,32,34−39^ in Table 1, it can be seen that the anhydrate and hexahydrate of both salt hydrates have the same unit cell except for the size. This size difference can be explained by the difference in the anion radius between the bromide and chloride. Since both salt hydrates are calcium halide based and have near-identical crystal structures, similar hydration steps can be expected. This can be supported by the phase transition equilibria mentioned above,^20^ which indicate a tetra- and dihydrate like the CaCl_2_ has. CaCl_2_, as mentioned above, has a complex path-dependent hydration–dehydration with its trito- and monohydrate,^15,16^ and the hydration steps also change when in the confinement of a porous matrix.^40^

The goal of this study is to find the phase transitions of the lower CaBr_2_ hydrates. Therefore, the kinetic onsets and, where possible, equilibrium lines were determined at low water vapor pressures. The corresponding powder X-ray diffractograms and single-crystal structures were also established. Additionally, the hydration and dehydration steps of CaBr_2_ inside the pores of mesoporous silica gels are compared to those of the pure salt and its sister salt CaCl_2_.

## Materials and Experimental Methods

### Materials

Calcium bromide (CaBr_2_) was ordered from Alfa Aesar in the form of an x-hydrate (CaBr_2_·H_2_O). For use as a TGA sample, the salts were ground very lightly in a mortar preheated in a 160 °C oven to avoid deliquescence. A saturated solution of CaBr_2_ with demineralized water was made to synthesize the composites.

As matrices, mesoporous and amorphous SGs were used with different pore diameters, which will be called SG11 and SG6 because of the rounded measured average pore diameters determined in ref (41). The average pore sizes of all of the used matrices are given in Table 2. The SGs were ordered from Sigma-Aldrich, and all of them were used without any pretreatment.

**Table 2:** Weight of the Different Composites Made from Silica Gels (SGs) with an Average Pore Diameter in the Nanometer Range and Pore Volume of about a Cubic Meter per Gram and a Saturated Solution of CaBr_2_ are Given as the Dry Salt Content (ϕ) and the Ratio between the Two Dry Components

composites	abbreviation	pore *d*_avg_ [nm]	pore *V* [m^3^/g]	ϕ [wt %]	*m*_salt_/*m*_SG_
SG11 + CaBr_2_	SG11CB	11.0	1.04	51.72	1.07
SG6 + CaBr_2_	SG6CB	6.0	0.79	43.96	0.78

### Synthesis of the Composites

**Figure 1:**
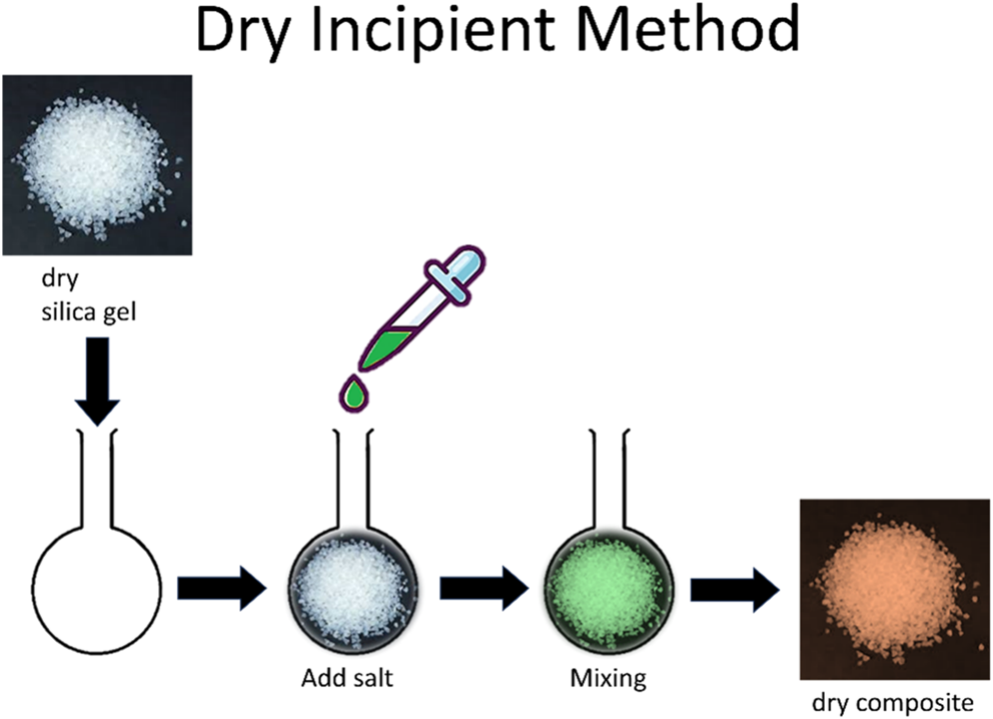
Schematic representation of the composite synthesis using the dry incipient method for the silica gels.

The silica gel composites were synthesized through the dry incipient method.^42−46^ The process of the dry incipient method, illustrated in Figure 1, involved drying matrices in an oven at 160 °C overnight to determine their dry weight. Subsequently, dried silica gels were mixed with a saturated aqueous salt solution with an amount that could fill the whole accessible pore volumes. The two components were mixed until resembling a dry powder due to the capillary forces sucking the solution into the pores. The composites were then dried overnight in an oven at 160 °C.

Through weighting the samples at various steps in the procedures, the masses (m) of the two components, the salt content (ϕ), and different ratios could be determined, as shown in Table 2. Here, the ϕ was calculated as

Equation 1—Salt content calculation of the dry salt in dry silica gel.
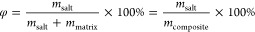


### Pore Characterization

The pore structure of the matrices was investigated by N_2_ adsorption and desorption isotherms at 77 K measured with a Micromeritics Gemini VII. First, the samples were prepared by degassing them in the preparation station at 150 °C with a N_2_ flow overnight (16 h). This measurement was done by isothermal adsorption and desorption at 77 K with pressures from 0 to 0.998 *p*/*p*_0_. By doing so, the average pore diameter, surface area, and accessible pore volume could be acquired. The Barrett–Joyner–Halenda (BJH) method^47^ was used to calculate the average pore diameter and determine the accessible pore volume, and the Brunauer–Emmett–Teller (BET) theory^48^ was used to determine the surface area in m^2^ per g of sample. The results for the silica gels used in this work are shown in Table 2 and ref (41)

### Metastable Behavior

Isobaric water sorption and desorption of the pure salts and the composites were investigated using Thermogravimetric Analysis on the two TGA devices as described in refs (16,41) Mettler Toledo TGA/SDTA851e and Mettler Toledo TGA/DSC 3+. The TGA setups were used together with a home-built or a Cellkraft humidifier. The oven temperature could be controlled between 25 and 1000 °C, and this temperature was recorded together with the sample temperature. The sample was located in both devices on a balance arm inside the oven and had an accuracy of ±1 μg.

Both machines had an inlet for gas flow connected to one of the humidifiers. The home-built humidifier operated at 18 °C and mixed a dry (0\% RH) and a wet (100\% RH) N_2_ flow to generate a water vapor pressure between 0 and 20 mbar. This was performed by Arduino-controlled flow meters, which could mix the two flows in different ratios to create the desired water vapor pressure. This home-built device was connected to the TGA/SDTA851e. The second humidifier was a Cellkraft Humidifier P2 operating at 25 °C, which worked via a feedback loop from an RH sensor at the outlet of the humidifier and was connected to the TGA/DSC 3+. Both devices had a flow rate of 300 mL/min over the sample inside the TGAs.

The temperatures of both TGA’s were calibrated to an accuracy of 0.2 K using the melting points of naphthalene, indium, lead, and zinc, which give a differential signal during this endothermic process,^49^ while the humidifiers were calibrated to an accuracy of $\pm$ 1 mbar using the gravimetric signal at the deliquescence point of LiCl·H_2_O, CH_3_COOK, K_2_CO_3_·1.5H_2_O, MgCl_2_·6H2O and Mg(NO_3_)_2_·6H_2_O at 25 °C and a validity check at higher temperatures (45 and 60 °C) using LiCl·H_2_O.^50^

The temperature programs were run with a sample and the humidifier simultaneously at the desired water vapor pressure. Thereby, the program was usually structured to contain an isothermal step at a high temperature to ensure complete dehydration, then cooling at a certain K/min rate, an isothermal step at the lowest temperature to ensure full hydration, and then heating at the same rate as the cooling back to the high temperature, which was again held in an isothermal step to ensure full dehydration again. The resulting weight changes were used to calculate a parameter called loading (L), which describes the weight change of the sample in [mol H_2_O per mol salt]. The loading was calculated similarly to ref (9) using the dry weight (*m*_d_ [mg]) at high temperatures of each sample and the current weight (*m* [mg]) together in below equation:

Equation 2—Loading calculation
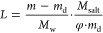
Here, *M*_w_ [g/mol] is the molecular mass of water (18.01528 g/mol), and *M*_salt_ [g/mol] is the molecular mass of the salt, calcium(II) dibromide anhydrate (199.89 g/mol). Lastly, ϕ represents the weight content of the dry salt in the sample as described in equation 1, which is between 0 and 100% for the composites and equal to 100% for the pure salt samples.

### Sorption–Desorption Equilibrium

The equilibrium vapor pressure–temperature lines of the CaBr_2_ transitions were measured using a so-called pT-meter, previously described in refs (9,11). This in-house-built setup consists of the Pfeiffer Vacuum CMR 361 pressure sensor with a range from 0.01 to 1100 mbar and a precision of 0.2%, a sample chamber with a heating mantle, and connecting tubing, which can be heated by a heating wire, with valves. The sample was prepared inside an aluminum pan by mixing the two hydrates around the desired equilibrium line. The specific hydrates were synthesized from the ordered x-hydrate in ovens at different temperatures, together with the humidity present under ambient conditions. Before the start of a measurement, the whole setup with valve 1 closed was evacuated for several minutes using the vacuum pump Edwards Oil Mist Filet EMF 10. This state was then used as the zero point in the control Matlab script. Subsequently, valve 1 was opened, and the sample chamber was evacuated for only a few minutes to prevent significant dehydration. Afterward, valve 2 was closed, and the measurement was started. The measurement consisted of steps of increasing the temperature. The sample was left to equilibrate for 6 h after each temperature step because then the measured pressure remained constant. The temperature range of the device was set from room temperature (around 25 °C) to 130 °C, with an additional step with a set value of 20 °C to compensate for an overshoot at the first heating of the heating mantle. During the measurement, the pressure inside the setup was measured as the difference between the zero point and the actual pressure. Since the sample was measured under vacuum, the salt hydrates equilibrate faster than under ambient conditions, and the measured pressure equals the equilibrium water vapor pressure. The device was leak-tested with helium and an empty measurement, which showed roughly 2.25 mbar leaking in per week of measuring. Since the measurement had durations from 2.75 to 3.25 days (maximum 0.9–1 mbar inleak) and was measured starting from low vapor pressures to around 20 mbar water vapor pressure at the end, this inleak is insignificant.

### Structural Characterization

Powder X-ray diffraction (PXRD) was performed by using a Rigaku Mini-Flex diffractometer in continuous scan mode with a divergent slit of 0.625° and a D/teX Ultra2 detector, using Cu Kα-radiation and K_β_ filter.^41^ To identify the crystalline phases of the confined salt hydrates and to observe the phase transitions, in situ PXRD was performed using a high-temperature attachment, called Anton Paar BTS 500 heating stage, built-in diffractometer, and attached humidifier, which can blow nitrogen with 0–20 mbar water vapor over the sample. The measurement was carried out with Bragg–Brentano geometry at 2θ = 3–90° with step sizes between 0.005 and 0.01° and a speed of 1 to 10 °/min. The humidifier worked similarly to the home-built humidifier of the TGAs, but the flow rate was set to 800 mL/min because of larger sample sizes.

Measurements were performed at different temperatures with a constant *p*_vap_ of 12 mbar. To minimize measurement time during in situ measurements, which required 30–50 scans, the scan conditions were carefully optimized. Considering that the strongest reflection of the anhydrate and different hydrates lie between 10° and 50°, the range of 2θ was set to 10–50° with a step size of 0.050° and a speed of 5°/min, which had a recording time per scan of 9 min. First, the sample was brought to a starting temperature of 170 °C when the first scan was recorded. Then, the temperature was decreased by 1 K/min, which was interrupted by steps every 2 K from 132 to 84 °C. A diffractogram was recorded at each temperature step. Then, the diffractogram at the lowest temperature of 84 °C was measured with a subsequent increase in temperature from 100 to 122 °C and from 140 to 162 °C in steps of 2 K and end temperature of 170 °C. The start, lowest, and end temperatures were held for 3 h before the reflections were recorded to ensure that the sample was completely transitioned. In contrast, all other temperatures were only held for 1 min before recording the reflections for an experiment similar to the TGA measurements.

### Single-Crystal X-ray Diffraction (SCXRD)

The crystal structures of the CaBr_2_ monohydrate and dihydrate single crystals were determined by performing SCXRD on the crystals grown in an autoclave as described above. A similar procedure was followed in ref (16) to make CaCl_2_ single crystals following the phase diagram of Sinke et al.^51^ However, no such phase diagram of the solubilities was found for CaBr_2_. Similar to the deliquescence onset, it was assumed that the CaBr_2_ phase diagram would be similar to the CaCl_2_ one at lower temperatures due to it being more hygroscopic. So, mixtures of 1.5 and 3 mol H_2_O per mol CaBr_2_ were made to create single crystals of the monohydrate and dihydrate, respectively. For the monohydrate, the sample was heated in the autoclave under pressure of 50 bar N_2_ to 250 °C for two h and was then allowed to cool back down to room temperature. For the dihydrate, nearly the same procedure was followed, but the sample was only heated to 200 °C.

Before the measurements, the crystals were coated with oil to prevent hydration and dehydration of the salt. Reflections were observed on a Bruker D8 Quest diffractometer with a sealed tube and a Triumph monochromator (λ = 0.71073 Å). The software package used for the intensity integration was Saint (v8.40a).^52^ Absorption correction was performed with SADABS.^53^ The structures were solved with direct methods using SHELXT-2014/5.^54^ Least-squares refinement was performed with SHELXL-2018/3^55^ against all reflections. Non-hydrogen atoms were refined freely with anisotropic displacement parameters. Hydrogen atoms were placed in calculated positions or located in different Fourier maps. All calculated hydrogen atoms were refined with a riding model.

## Results and Discussion

### Hydration–Dehydration Steps and their Structures

**Figure 2:**
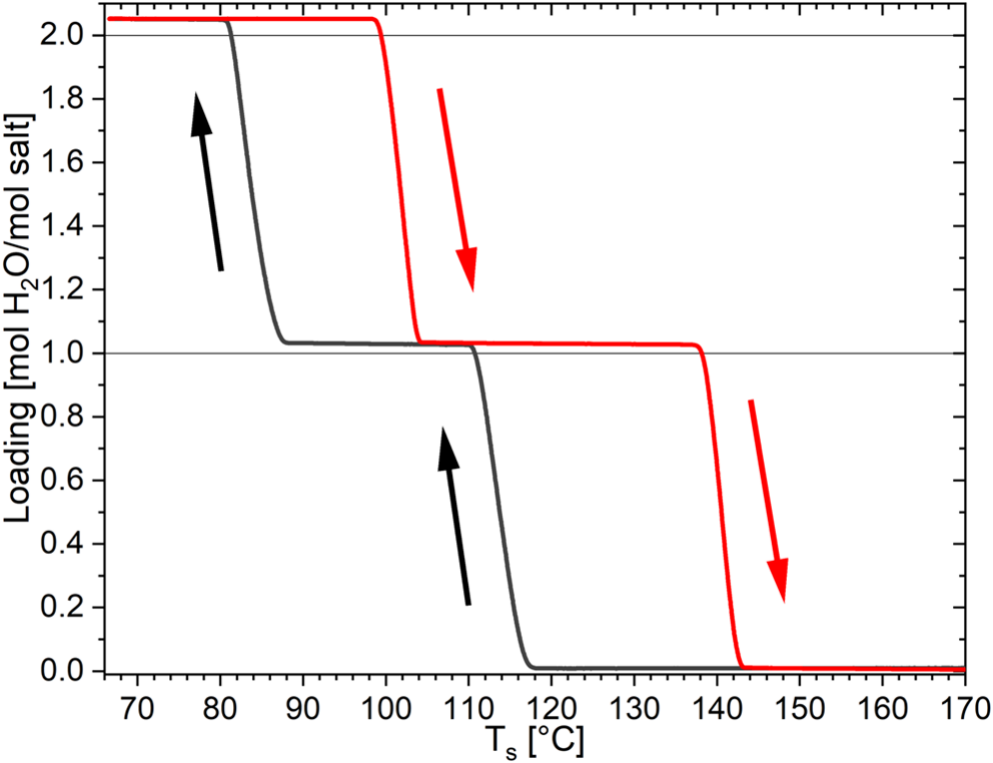
Isobaric TGA measurements of CaBr_2_ at 10 mbar water vapor pressure and a cooling/heating rate of 0.2 K/min. The cooling is the black curve, and the heating is the red curve.

The first question regarding CaBr_2_ is about the hydration and dehydration pathways. Therefore, two types of isobaric TGA measurements were performed with CaBr_2_: (1) multiple hydration–dehydration cycles at 1 K/min heating and cooling rate and (2) cycles of increasing heating/cooling rate from 0.2, 0.5, and 1 K/min. An example of the 0.2 K/min cycle at a water vapor pressure of 10 mbar is shown in Figure 2. The other cycles are given in the Supporting Information. In Figure 2, it is visible that when the temperature is decreased from the starting point at 170 °C, the weight of the salt remains constant until around 117 °C. At this temperature, 1 mol H_2_O per mol CaBr_2_ is taken up to form the monohydrate. The monohydrate is stable throughout the continuous cooling until around 88 °C. The sample takes water up to a loading of 2 mol/mol CaBr_2_. This is the last hydrate formed over the cooling part of the TGA measurement down to 66 °C. Over the subsequent heating part back to 170 °C, the same hydrates as during the cooling are formed again in reversed order (2–1–0 instead of 0–1–2), where the steps are around 99 and 137 °C.

The onset temperatures remained constant within the error margins across various heating/cooling cycles. The only difference observed is in the slope between the loading plateaus (see Figure S1). Similarly, in the multi cycles at 1 K/min experiment (see Supporting Information), the onset temperatures of the phase transition also remained at the same temperatures. Only the slope of the hydration/dehydration events increased over cycling, which implies an increase of the reaction kinetics over cycling, which is common for salt hydrates.^9,12,56^

Compared to the sister salt CaCl_2_, CaBr_2_ seems to behave far more regularly. For CaCl_2_, it was found that the hydration and dehydration steps are path-dependent. Additionally, at 1 K/min, the steps of CaCl_2_ during hydration are not yet stable until after 10 cycles and keep changing with each subsequent cycle.^16^ This shows that similar salt hydrates, such as CaBr_2_ and CaCl_2_, with similar crystal structures for the anhydrate and hexahydrate, do not form the same hydrate steps.

Here, the different hydrates were determined only by the weight change in the sample represented as loading [mol H_2_O/mol CaBr_2_]. But are these single hydrates or mixtures of multiple hydrates and is the intermediate during hydration the same as during dehydration? PXRD in situ measurements were performed to support the assumption from the TGA weight changes.

**Figure 3:**
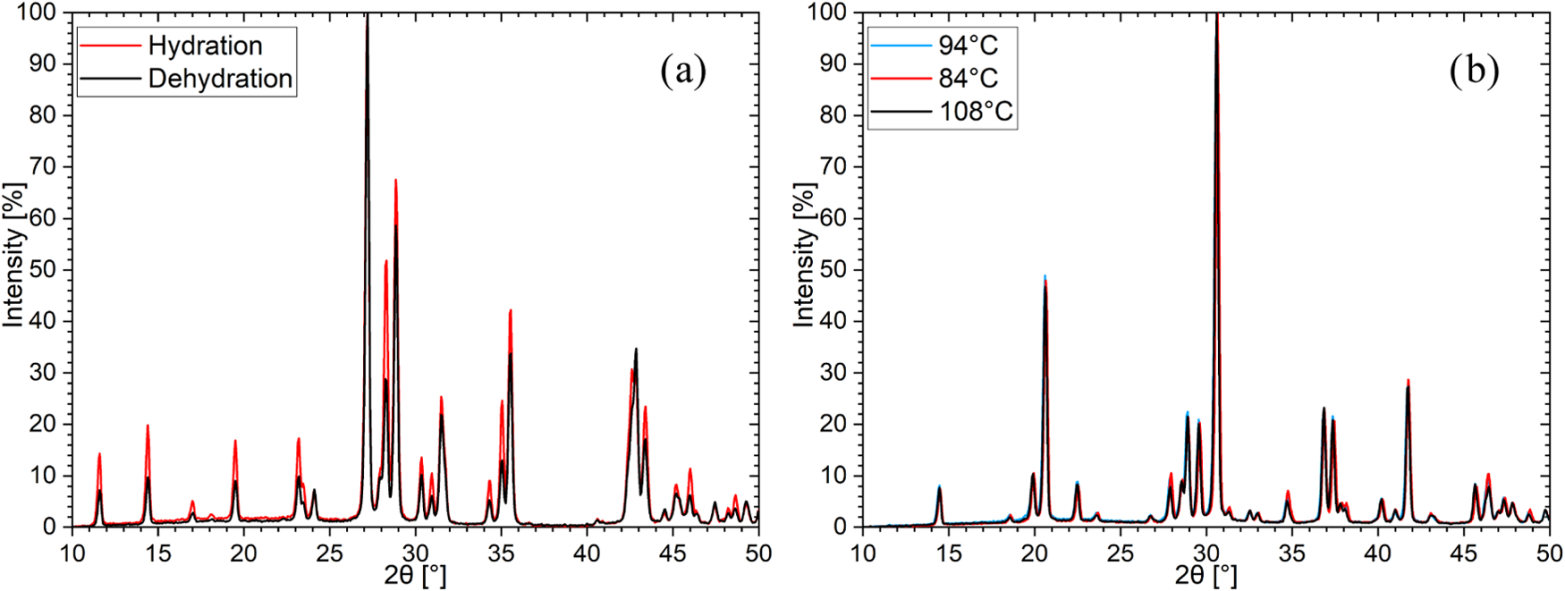
Isobaric PXRD in situ measurements of CaBr_2_ at 12 mbar as (a) monohydrate during cooling at 116 °C (red) and heating at 148 °C (black) and (b) dihydrate at its first formation (94 °C, blue), at the lowest temperature of the measurement (84 °C, red), and before the dehydration to the monohydrate (108 °C, black).

Three different phases could be identified during both hydration and dehydration: phase A (anhydrate), phase I (intermediate), and phase H (hydrate). These three phases can be distinguished by their most prominent reflections: phase A at 2θ of 28 and 37°, phase I at 2θ of 27, 29, and 35.5° (Figure 3a), and phase H at 2θ of 20.5 and 30.5° (Figure 3b). The initial phase during hydration (and the final phase during dehydration) could be determined as the anhydrate, according to ref (31). According to the TGA measurements, the second phase appearing during hydration was assumed to be the monohydrate. Additionally, this assumed monohydrate appears as the intermediate during the dehydration, like in the TGA results. During the PXRD in situ measurements, the monohydrate was observed under conditions (temperatures and water vapor pressures) similar to those for the TGA results. The diffractograms of the assumed monohydrate are shown in Figure 3a, with one of the hydration diffractograms in red and one of the dehydration ones in black. According to the TGA results, the third phase appearing during hydration at the lowest temperatures (the first phase during dehydration) is assumed to be the dihydrate and is shown in Figure 3b. The reflections with at least 10% intensity of the most intense reflection for each of the two hydrate phases are listed in a table in the Supporting Information. However, no planes could be assigned to these reflections since no crystal structures of the mono- and dihydrate could be found in the COD, ICSD, CCDC, ICDD PDF-4+, and AMCSD databases. Even so, it could be shown that three sets of reflexes correlate with the three plateaus observed during the TGA measurements, which supports the assumption that the mono- and dihydrate are formed instead of mixtures of hydrates.

**Figure 4:**
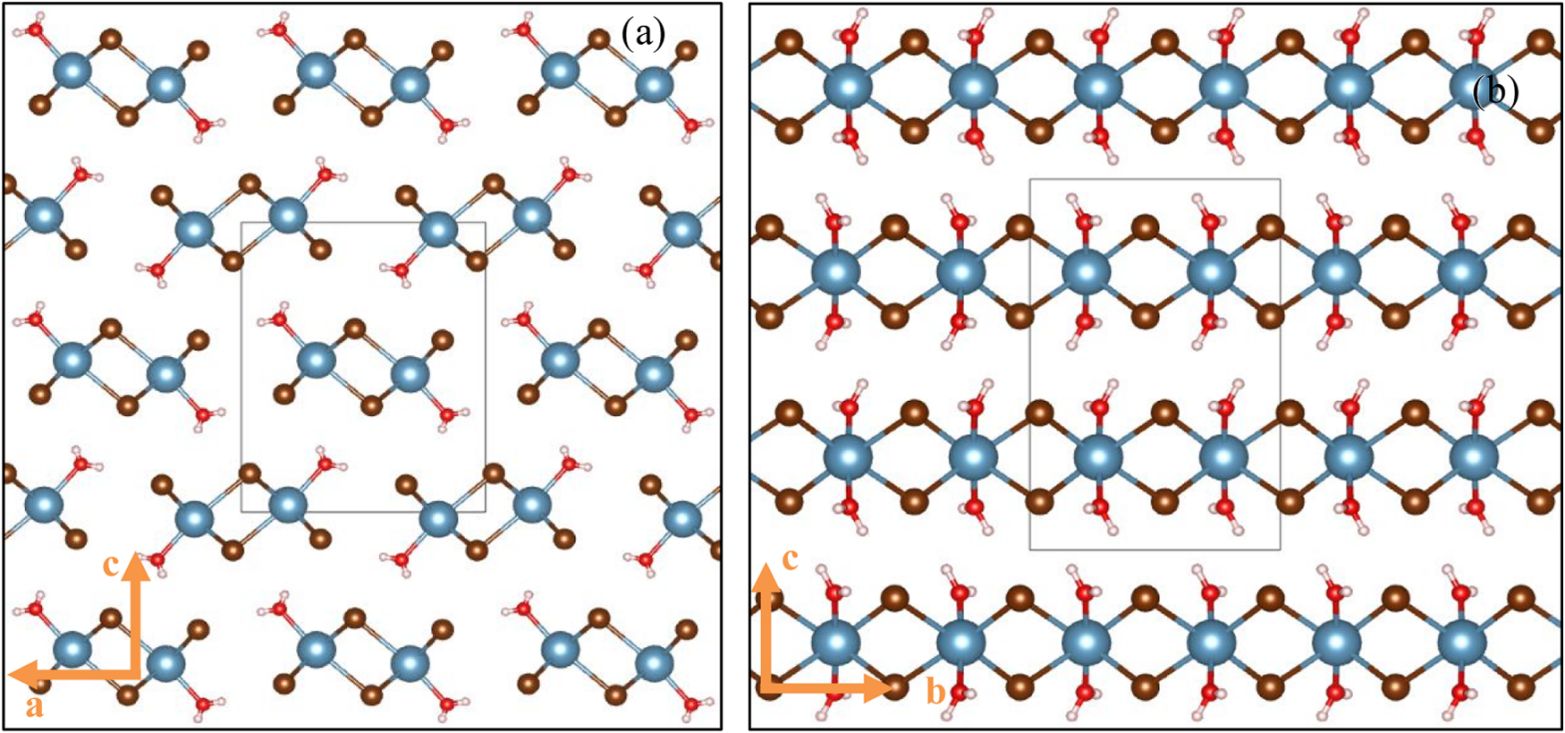
Single-crystal structures of the CaBr_2_ (a) monohydrate and (b) dihydrate, as determined in this work. The blue spheres represent the Ca^2+^ ions, the brown ones the Br ions, the red ones the oxygens, and the white ones the hydrations.

To confirm the structure of the different crystalline phases formed during the hydration–dehydration cycle, single crystals of the monohydrate and dihydrate were grown and analyzed with SCXRD. The results of the SCXRD analysis showed that the dihydrate has nearly the same crystal structure as the CaCl_2_ dihydrate (see Table 3), which is a *Pbcn* symmetry (see Table 3). Despite that, the unit cell volume of 596.28 Å^3^ was slightly bigger than its chloride counterpart due to the size difference of the anions in the salt. The monohydrate has the space group *Pnma*, which differs from the CaCl_2_ monohydrate with the space group *Pmmm*. Furthermore, CaBr_2_ monohydrate forms columns from stacks of two calcium ions connected by their ligands. A picture of the two newfound crystal structures is shown in Figure 4. The simulated PXRD diffractograms of both crystal structures were compared to the diffractograms of the PXRD in situ measurement and were found to overlap. This proves the assumption of the monohydrate and dihydrate steps of both isobaric measurements (TGA and PXRD).

**Table 3:** Unit Cells of the CaCl_2_ and CaBr_2_ Hydrates Taken from refs (16,30−31,32,33,34,39) and Determined in This Work

	CaCl_2_			CaBr_2_		
hydrate	space group	symmetry	*V* [nm^3^]	space group	symmetry	*V* [nm^3^]
anhydrate	*Pnnm*	orthorhombic	0.169	*Pnnm*	orthorhombic	0.196–0.202
tritohydrate	*Pnma*	orthorhombic	0.943			
monohydrate	*Pmmn*	orthorhombic	0.185	*Pnma*	orthorhombic	0.507
dihydrate	*Pbcn*	orthorhombic	0.531	*Pbcn*	orthorhombic	0.596
α-tetrahydrate	*P*-1	triclinic	0.331			
β-tetrahydrate	*P*2	monoclinic	1.060			
γ-tetrahydrate	*P*2	monoclinic	0.391			
hexahydrate	*P*321	trigonal	0.212	*P*321	trigonal	0.232
nonahydrate				P21	monoclinic	1.229

Unlike the two intermediate phases of CaCl_2_, which deviated from the typical octahedral coordination of six ligands around each Ca^2+^ ion by adopting a capped trigonal prismatic molecular geometry or a squared antiprismatic one with seven or eight ligands, CaBr_2_ consistently maintained a strict adherence to six ligands across all crystal structures, ranging from the anhydrate to the hexahydrate, maintaining octahedral coordination. This could be a result of the larger ionic radius of bromide (180 pm) compared to chloride (165 pm),^57^ which hinders rearrangements to other coordinations than the octahedral one or does not allow more than six ligands due to space limitations around the Ca^2+^ ion.

### Phase Diagram

**Figure 5:**
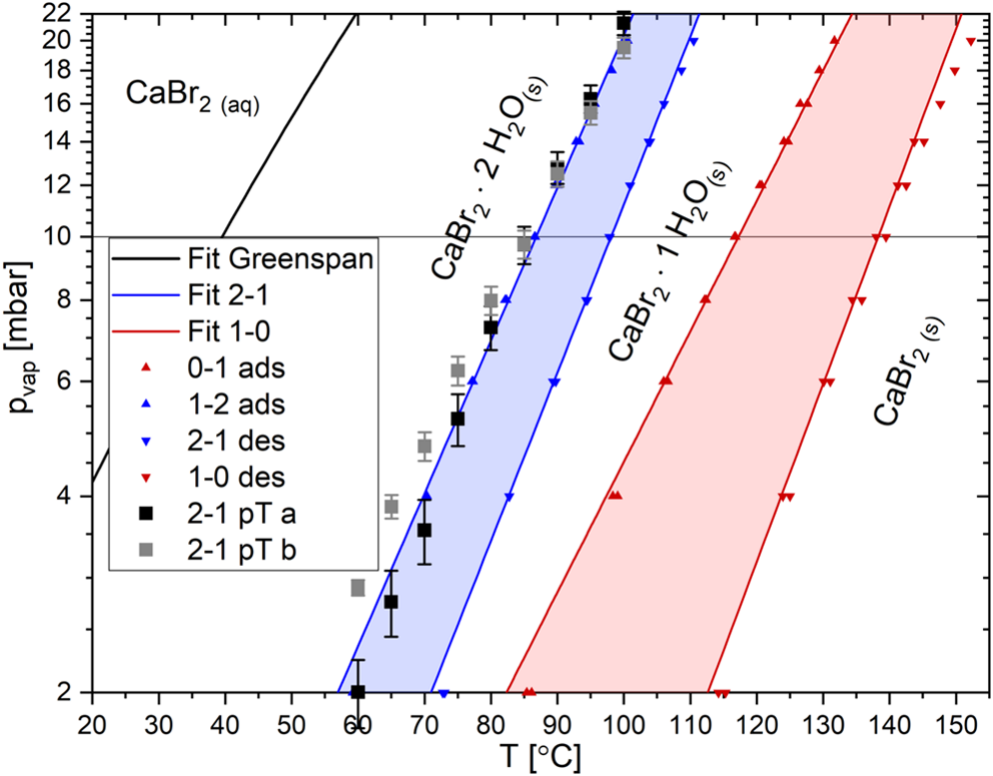
Phase diagram of the CaBr_2_ established from the kinetic measurements with isobaric TGA experiments as the triangles with red for the 0–1 and blue for the 1–2 transition and equilibrium line measurements with the pT-meter as the squares.

Now that the hydration and dehydration steps of CaBr_2_ are determined by their weight and connected to their crystal structures, the phase diagram of these transitions can be derived. For this, the deliquescence relative humidity (DRH) of CaBr_2_ determined by Greenspan^50^ at 10, 15, 20, and 25 °C to be at 21.62–16.50% relative humidity (RH) was used to indicate the onset of this solid–liquid phase transition. A fit through the four values provided by Greenspan is shown in Figure 5 as a black line.

The kinetic onsets at different water vapor pressures were determined for the solid–solid phase transitions during hydration and dehydration. Therefore, the TGA measurements of hydration–dehydration cycles were repeated at water vapor pressures of between 2 and 20 mbar. The onset temperatures of the four different phase transitions are plotted in Figure 5 as the triangle symbols. The upward-pointing triangles represent the hydration onsets (taking up water) and the downward triangles correspond to the dehydration onsets (losing water). The red symbols are for the 0–1 and 1–0 phase transitions and the blue ones are for the 1–2 and 2–1 phase transitions. A fit was done through the different phase transition onsets represented as the red and blue lines according to the hydration steps (0–1 and 1–0 vs 1–2 and 2–1).

The kinetic onsets of the two-phase transitions form a region of slow kinetics called the Metastable Zone (MSZ). The width of the 0–1 MSZ is wider than the 1–2 MSZ. This is similar to the observations done by Sögütoglu et al. for MgCl_2_^11^ and Blijlevens et al. for SrCl_2_.^12^

In addition to the kinetic onsets, measurements were performed with the pT setup. The 1–2 equilibrium line results are depicted in Figure 5 as the black and gray squares. The equilibrium line is very close to the 1–2 hydration onsets. This indicates that CaBr_2_ has no hydration MSZ for the 1–2 transition, while it has one for the dehydration. The 1–2 MSZ and equilibrium line of CaCl_2_ and the 0–1 MSZ and equilibrium line of LiCl^11^ and LiBr^10^ and own measurements shown in the Supporting Information have similar behavior. This suggests that there is no nucleation barrier^9^ for the formation of CaBr_2_·2H_2_O from CaBr_2_·H_2_O.

The 0–1 phase transition could not be measured, as it was outside the reliable measurement range of this pT setup. Hence, no comparison between the kinetic onsets and the equilibrium line of this 0–1 transition can be made. Nonetheless, the MSZ of 0–1 and 1–0 is wider than for the 1–2 transition, indicating that the nucleation barrier is or the nucleation barriers are more pronounced for the lower hydrate (0–1 and/or 1–0) than for the transitions to and from the dihydrate.

### Composites

**Figure 6:**
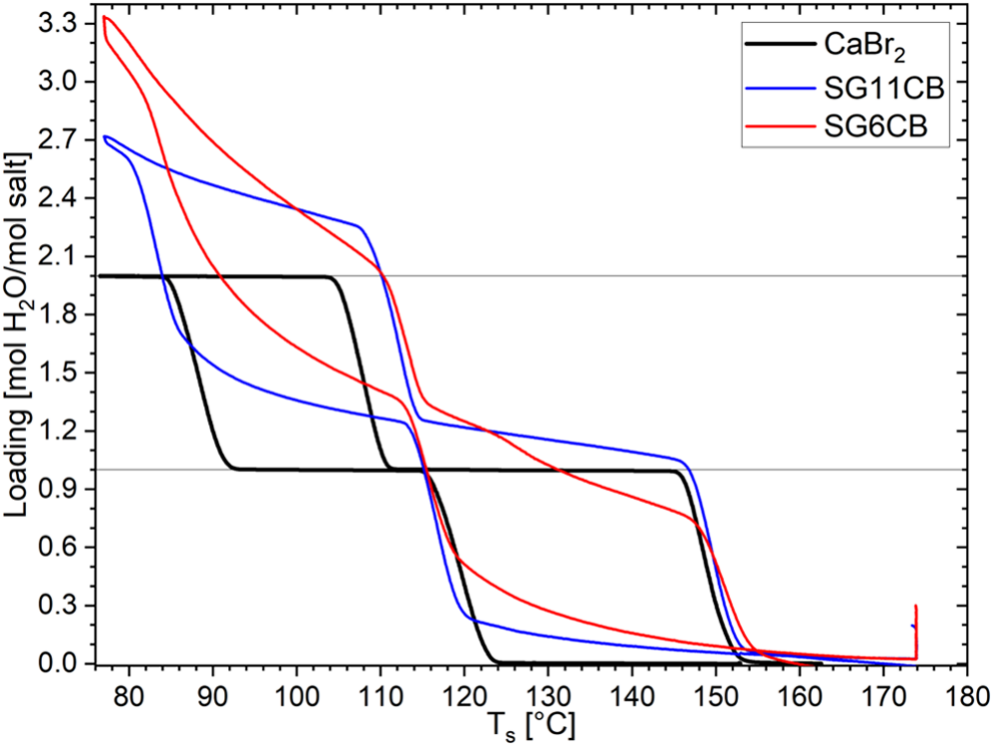
Results of isobaric TGA experiments at 12 mbar water vapor pressure and 0.5 K/min temperature ramps of the composites SG6CB in red and SG11CB in blue with the pure CaBr_2_ salt as a reference in black.

During the studies on CaCl_2_ in refs (16,40), it was found that the path-dependent hydration–dehydration steps can change under confinement. To examine if similar changes are happening in the hydration–dehydration steps of CaBr_2_, two silica gels (SGs) with average pore diameters of 6 and 11 nm were impregnated with CaBr_2_. N_2_ sorption showed that pore volumes are 1.042 m^3^/g for SG11 and 0.792 m^3^/g for SG6.^41^ These composites are called SG6CB and SG11CB. These composites were then subjected to similar isobaric TGA cycles at 12 mbar as the pure salt described above. The results of these 12 mbar measurements are shown in Figure 6, together with the pure salt measured at the same water vapor pressure and temperature ramp.

In Figure 6, it is visible that at the lowest measured temperatures, the composites have a significantly higher loading than the pure salt (2.7 for SG11CB and 3.3 for SG6CB). This is most likely the result of the adsorption of water vapor onto the surfaces of the silica gel matrices, as described in ref (41). It is not assumed that deliquescence of the salt hydrate is involved because clear dehydration transition onsets are observed and no significant uptake is observed at the lowest temperature between cooling and heating.

When examining the blue line for SG11CB in Figure 6, you can observe an onset from the anhydrate to the monohydrate around 121 °C, followed by a gradual transition from the monohydrate to the dihydrate around 87 °C. Both are very similar to the onsets of pure CaBr_2_, as shown in the black line and the values given in Table 4, together with the fit lines from the phase diagram in Figure 5. The same is true for the 1–0 dehydration onset. However, the 2–1 dehydration onset is slightly shifted to higher temperatures, which might be the result of the SG adsorption or deliquescence of the salt, making it more difficult to release the water from the sample. The difference in onset temperature in Table 4 is only 3 K, which is insignificant compared to the deviation of the pure salt with the fit lines.

**Table 4:** Onset Temperatures [°C] at 12 mbar of Isobaric Hydration–Dehydration Measurements of CaBr_2_, SG11CB, and SG6CB Presented in Figure 6

	0–1	1–2	2–1	1–0
fit line phase diagram	121	90	101	141
CaBr_2_	124	92	104	145
SG11CB	121	87	107	146
SG6CB	∼119	∼89	110	147

For the SG6CB composite, all onsets, especially the hydration ones, are smoothed and not as sharp as for SG11CB or pure salt. This is caused by the adsorption of the matrix, which is stronger for SG6 than for SG11, and the pore size distribution creates a spread in crystal sizes. However, the onsets of SG6CB, as far as we can identify, are also the same as the ones of the SG11CB composite and mostly the same as the pure salt. This correlates well with earlier findings with single-hydrate salts in silica gels,^41^ that the crystal size or the adsorption from a host matrix does not change the kinetic (de)hydration onsets. This could mean that even for salt hydrates with multiple hydrate steps like CaBr_2_, the confinement by a host matrix does not influence the kinetic hindrances for the hydration and dehydration onsets.

Even though the 2–1 dehydration onset of the composites differs slightly from that of the pure salt, the observed steps are the same as in the bulk CaBr_2_ (0–1–2–1–0). This can also be supported by isobaric PXRD in situ measurements of these composites in comparison with the pure salt presented in the Supporting Information. So, while the CaCl_2_ composites had different hydration steps depending on the matrix used/the pore size in the matrix and also different from those of the pure CaCl_2_ (0–1/3–2–1–0),^40^ for the CaBr_2_ composites this is not the case.

The main differences between these two Ca salts are the intermediate steps between the anhydrate and dihydrate. For CaBr_2_, there is only the monohydrate, which has clearly defined conditions when it is stable and does not overlap with another hydrate under those conditions, as shown in Figure 5. CaCl_2_, on the other hand, has two intermediate hydrates, trito- and monohydrate, that are kinetically hindered compared to the formation of the other.^15,16^ During hydration, CaCl_2_ follows a 0–1/3–2 pathway and skips the monohydrate. During dehydration, it follows a 2–1–0 pathway and skips the 1/3 phase. This indicates that the 1/3–1 transition is kinetically hindered. On the contrary, CaBr_2_ demonstrates nicely reversible behavior regarding its hydration and dehydration pathways.

## Conclusions

In this study, the hydration and dehydration paths of CaBr_2_ were investigated on their hydrate steps together with the corresponding crystal structures and temperature-water vapor pressure phase diagram. Additionally, the hydrate steps in the confinement of the silica gels were examined. It was found that independent of the number of cycles done before, the speed of temperature change, and whether it was as bulk salt or in a composite, CaBr_2_ always showed the hydration–dehydration from the anhydrate via the monohydrate to the dihydrate and back in reverse order (0–1–2–1–0).

This reversibility of the hydration steps could be confirmed by PXRD in situ. Additionally, the diffractograms of the monohydrate and dihydrate could not be linked to any known crystal structure. The structures determined with SCXRD showed that the CaBr_2_ only formed octahedral coordinations for its anhydrate, monohydrate, and dihydrate. This differed from the hydrate steps and structure found for CaCl_2_, which forms a path dependently on the trito- or monohydrate with more than six ligands surrounding the Ca ion. The difference in the monohydrate coordination could also be the result of the larger ion radius of the bromide, limiting the number of ligands to six, compared to the chloride with more flexible ligand coordination/number.

By repeating the hydration–dehydration cycles under different conditions, the MSZs of the 0–1 and 1–2 transitions in the phase diagram could be determined together with the equilibrium line for the 1–2 transition. Here, the position of the equilibrium line in the 1–2 MSZ showed that there is (nearly) no nucleation barrier during hydration, while this is the case for dehydration. No similar comparison could be made for the 0–1 transitions due to limitations in the setup.

Furthermore, the pathways in silica gel composites were tested in comparison with those of the pure salt, which showed that the same hydrate steps were formed under confinement as in the bulk salt. This differs from the findings for CaCl_2_, which had different steps depending on the pore size due to changes in the kinetic hindrance between phase transitions.^40^ In the hydration–dehydration cycles of CaCl_2_, it was found that the steps are path-dependent because the hydration (0–1/3–2) differs from the dehydration (2–1–0).^15^ The reason behind this path dependency is probably a hindrance of the 1/3–1 transition kinetics compared to the kinetics of the 1/3–2 hydration and 1–0 dehydration. Through the lower flexibility in crystal structures of the CaBr_2_, no kinetic hindrance between hydrate was present in the pure salt or composite, leading to unchanged hydration–dehydration pathways, as observed for single-hydrate salts in composites.^41^ This predictable and reversible hydration and dehydration behavior in and outside the confinement of a porous host matrix can make CaBr_2_ a good candidate for thermal energy storage.

## Supporting Information Available

Different TGA measurements of CaBr_2_ are shown, which were performed as multiple cycles with the same heating/cooling rate and as cycles at different heating/cooling rates; a list of the PXRD reflections of the mono- and dihydrate with a relative intensity of 10% or more is given to identify the CaBr_2_ hydrates during in situ measurements or for fingerprinting; PXRD in situ measurements on the bulk salt and the silica gel composites in this work are given as contour figures; the raw data of an equilibrium line of the CaBr_2_ 2–1 transition is shown; phase diagram of the salt hydrate LiBr, which was referenced in this manuscript, is given. (PDF)
