# Supplementary material for: Understanding Hydration Transitions of CaBr2
Source: Cryst Growth Des. 2025 Mar 27;25(8):2409–17. doi: 10.1021/acs.cgd.4c01522 (PMC12006962; doi:10.1021/acs.cgd.4c01522)
Supplement: Supplementary file 1 — cg4c01522_si_001.pdf [file cg4c01522_si_001.pdf]

# Supporting Information for "Understanding Hydration Transitions of $CaBr_2$"

*Michaela C. Eberbach1,2, Aleksandr I. Shkatulov1,3, Paul Tinnemans4, Hendrik P. Huinink*1,2, Hartmut R. Fischer5 and Olaf C. G. Adan1,5*

1 Eindhoven University of Technology, Den Dolech 2, 5600 MB Eindhoven, The Netherlands

2 EIRES, Horsten 1, 5612 AX Eindhoven, The Netherlands

3 Iberian Center for Research in Energy Storage, CIIAE, Polígono 13, Parcela 31, "El Cuartillo", 10004 Cáceres, Spain

4 Radboud University, Houtlaan 4, 6525 XZ Nijmegen, The Netherlands

5 TNO Materials Solutions, High Tech Campus 25, 5656 AE Eindhoven, The Netherlands

## Description

Different TGA cycles of $CaBr_2$ from anhydrate to dihydrate

multiple cycles at the same temperature-changing rate

cycles at different temperature-changing rates

List of PXRD reflections of the mono- and dihydrate with a relative Intensity of 10 % or more

PXRD in situ results of $CaBr_2$ and the composites of $CaBr_2$ + silica gel

pT-meter raw data of the 2-1 equilibrium line

The LiBr phase diagram, which was referred to in the manuscript

Bibliography

## Different TGA cycles

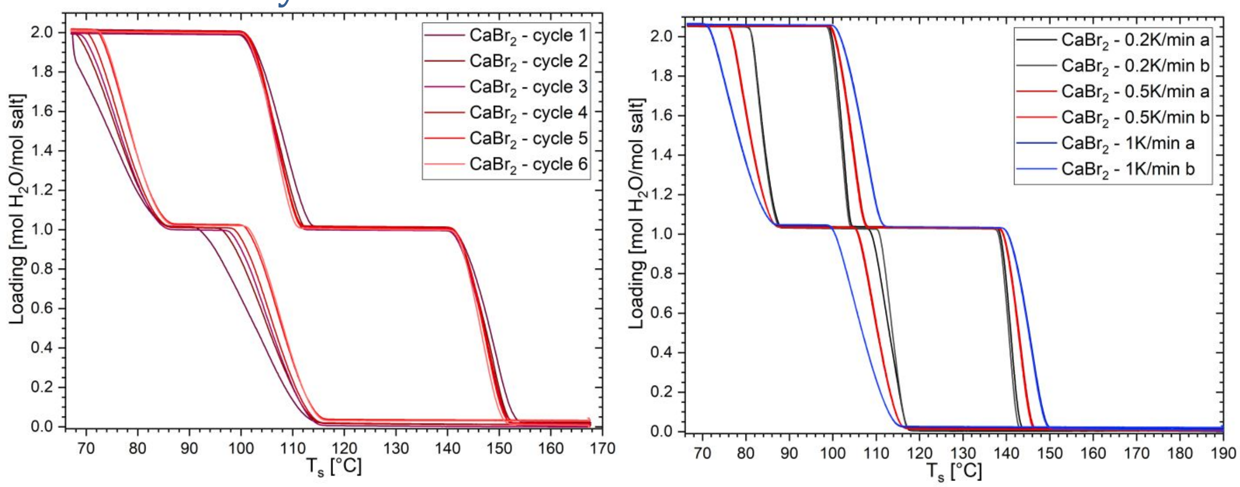


Figure 1 – The isobaric TGA measurements at 10 mbar water vapor pressure of $CaBr_2$ as a) cycles on one sample with 1K/min rates and b) fresh samples measured with different rates of 0.2-1 K/min.

# PXRD reflections of Mono- and Dihydrate

| Monohydrate | | | | | Dihydrate | | | | |
|---|---|---|---|---|---|---|---|---|---|
| 2θ [°] | θ [°] | d [nm] | Intensity [%] | plane (h k l) | 2θ [°] | θ [°] | d [nm] | Intensity [%] | plane (h k l) |
| 11.60 | 5.80 | 0.76 | 13.94 | (1 0 1) | 19.95 | 9.98 | 0.44 | 10.11 | (1 1 1) |
| 14.40 | 7.20 | 0.61 | 19.14 | (0 0 2) | 20.65 | 10.33 | 0.43 | 47.81 | (1 0 2) |
| 19.50 | 9.75 | 0.45 | 16.56 | (2 0 1) | 27.95 | 13.98 | 0.32 | 10.46 | (1 2 1) |
| 23.20 | 11.60 | 0.38 | 16.85 | (2 0 2) | 28.95 | 14.48 | 0.31 | 21.63 | (0 0 4) |
| 27.20 | 13.60 | 0.33 | 100.00 | (1 1 2) | 29.61 | 14.80 | 0.30 | 19.81 | (2 0 0) |
| 28.30 | 14.15 | 0.32 | 52.26 | (3 0 1) | 30.65 | 15.33 | 0.29 | 100.00 | (1 2 2) |
| 28.85 | 14.43 | 0.31 | 65.64 | (2 1 1) | 36.90 | 18.45 | 0.24 | 22.51 | (0 2 4) |
| 30.35 | 15.18 | 0.29 | 13.93 | (0 0 4) | 37.45 | 18.73 | 0.24 | J20.60 | (2 2 0) |
| 30.95 | 15.48 | 0.29 | 10.33 | (3 0 2) | 41.75 | 20.88 | 0.22 | 28.57 | (2 0 4) |
| 31.50 | 15.75 | 0.28 | 24.38 | (2 1 2) | 46.45 | 23.23 | 0.20 | 1-.26 | (3 1 0) |
| 35.05 | 17.53 | 0.26 | 24.40 | (3 0 3) | | | | | |
| 35.55 | 17.78 | 0.25 | 40.71 | (3 1 1) | | | | | |
| 42.60 | 21.30 | 0.21 | 29.11 | (0 1 5) | | | | | |
| 42.85 | 21.43 | 0.21 | 29.23 | (4 1 0) | | | | | |
| 43.40 | 21.70 | 0.21 | 23.00 | (4 1 1) | | | | | |
| 46.00 | 23.00 | 0.20 | 11.08 | (3 0 5) | | | | | |

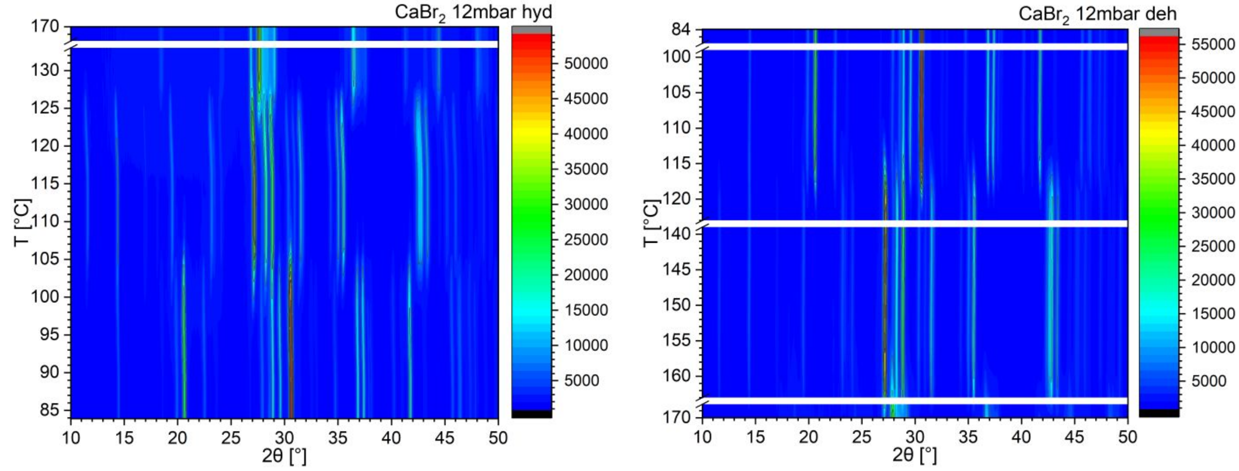


Figure 2 – The a) hydration and b) dehydration of pure $CaBr_2$ PXRD measurements at 12 mbar.

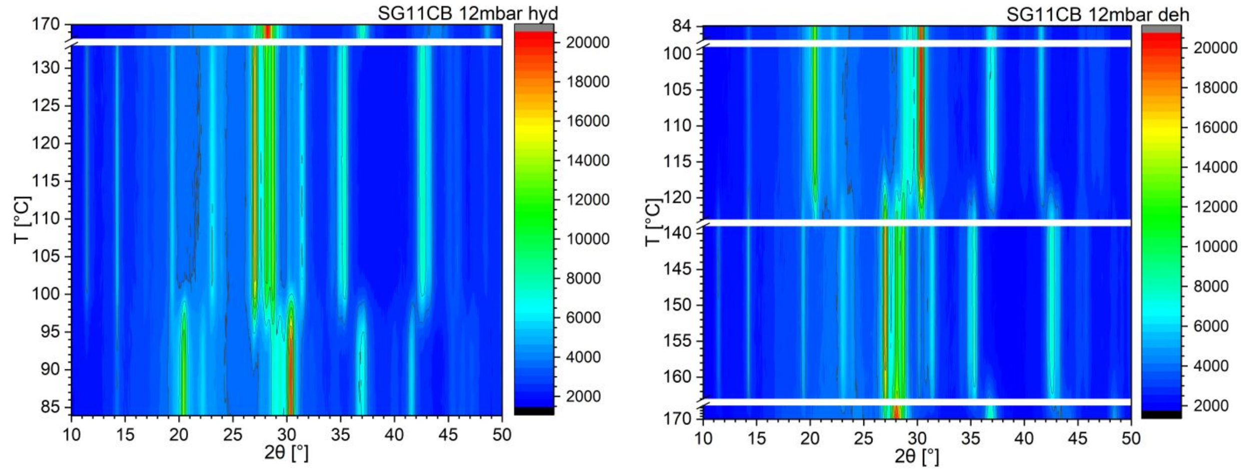


Figure 3 - The a) hydration and b) dehydration of SG11CB PXRD measurements at 12 mbar.

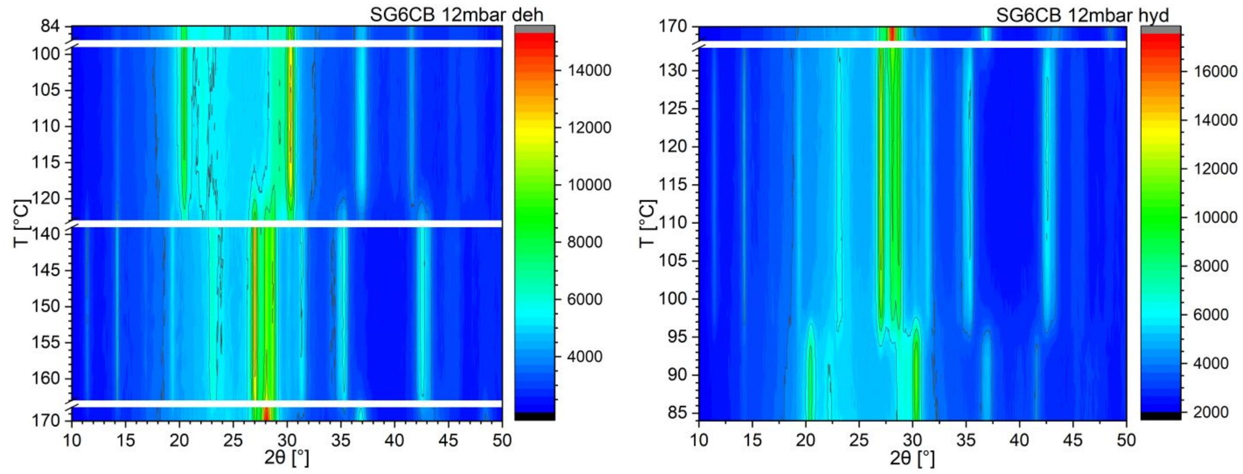


Figure 4 - The a) hydration and b) dehydration of SG6CB PXRD measurements at 12 mbar.

## pT-meter raw data

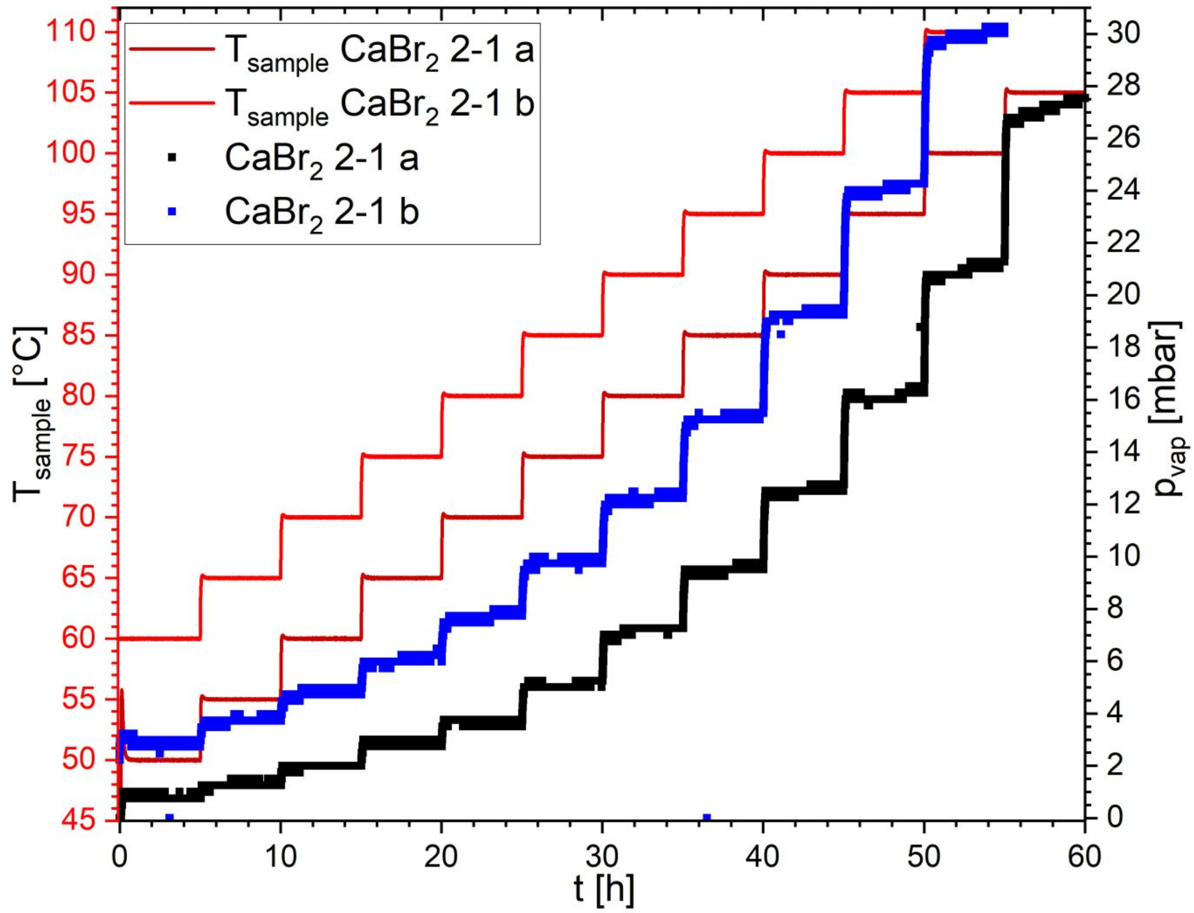


Figure 5 - The measurement results from the pT-meter of the $CaBr_2$ 1-2 equilibrium line.

## LiBr Phase Diagram

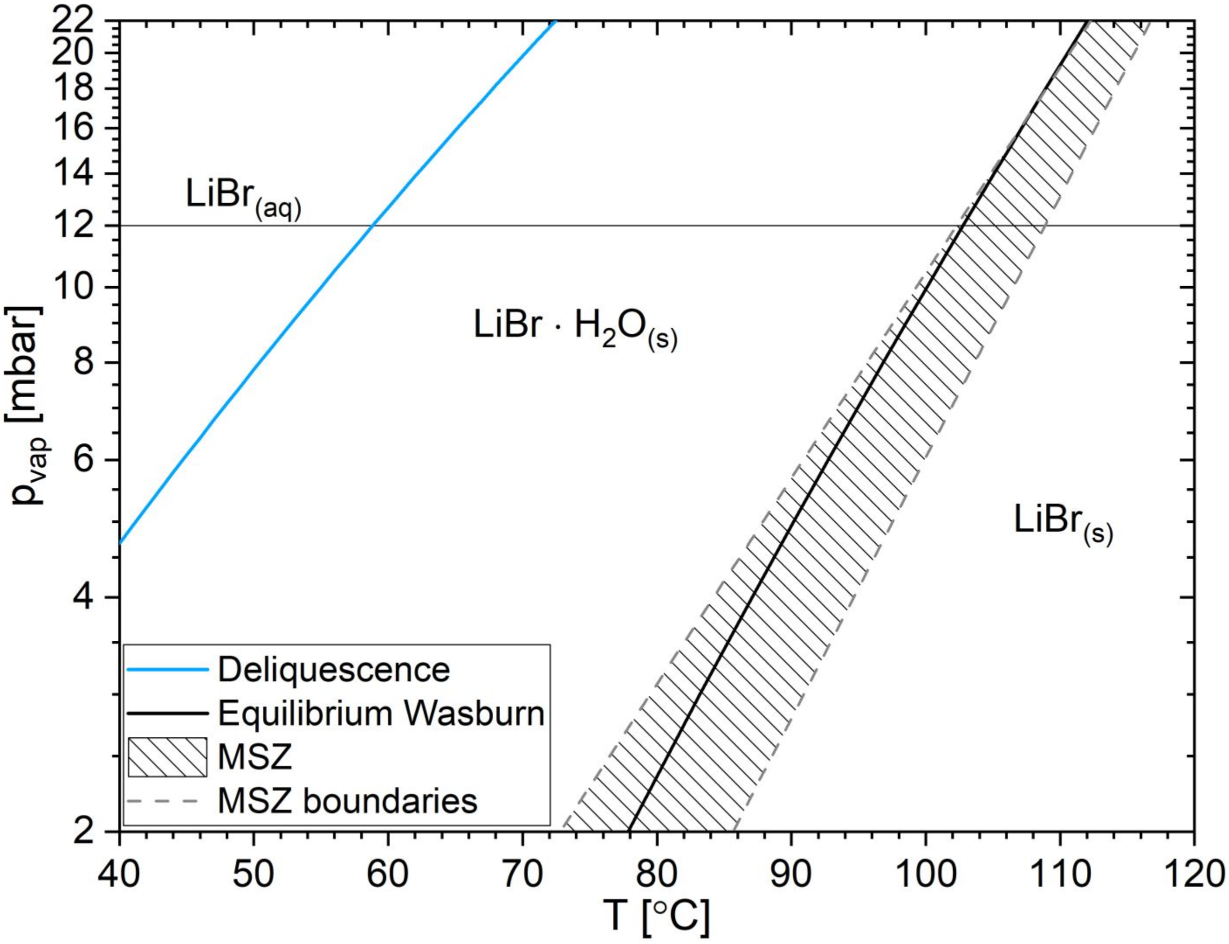


Figure 6 - The Phase Diagram of the LiBr established from the kinetic measurements with isobaric TGA experiments from the authors and equilibrium line from [1] and deliquescence onset line form [2].

# References

[1] E.W. Washburn, 1933, International critical tables of numerical data, physics, chemistry and technology: Volume III. (1928), Published for the National Research Council by McGraw-Hill.
[2] L. Greenspan, 1977, Humidity fixed points of binary saturated aqueous solutions, Journal of Research of the National Bureau of Standards - A Physics and Chemistry, Vol. 81A, pp. 89-96, DOI: https://doi.org/10.6028/jres.081A.011.
